# Collection Series “Iron Homeostasis”

**DOI:** 10.3390/ijms26188954

**Published:** 2025-09-14

**Authors:** Antonella Roetto, Paolo Arosio

**Affiliations:** 1 Department of Clinical and Biological Sciences, University of Turin, 10126 Turin, Italy; 2 Department of Molecular and Translational Medicine, University of Brescia, 25123 Brescia, Italy

Iron is essential for almost all living organisms, but excess or deregulated iron is potentially toxic [1,2] and can have severe health consequences [3,4]. Thus, many studies have been conducted on the (dys)regulation of iron homeostasis, uncovering the complex, regulated interplay of iron absorption, transport, utilization, and storage mechanisms [5]. We organized this Special Issue to collect new data on iron homeostasis. It attracted interest, and some interesting manuscripts were submitted and accepted for publication, including two review papers and three original research manuscripts addressing different aspects of iron metabolism. These manuscripts report noteworthy findings on topics that remain mostly unclear, such as the intricate control of iron metabolism in specific cell types and areas of the body and in the context of widespread health issues, as iron deficient anemia (IDA) and malaria. Notably, the majority of the articles in this Special Issue also address the role of iron metabolism in mammalian aging and neurodegenerative disorders, a significant medical issue [6,7] with considerable socioeconomical repercussions [8,9]. A crucial aspect of iron management that impairs the welfare of domesticated animals globally was also investigated.

Connor et al.’s interesting review [10] discusses the effects of common variants of the HFE gene that are known to contribute to systemic increases in iron levels. Mutations in this gene are responsible for autosomic recessive hereditary hemochromatosis [11], but their effects on iron homeostasis in the brain are unclear. In particular, there are conflicting data as to whether HFE variants protect against or exacerbate neurodegeneration [12,13]. The review focuses on the H63D HFE variant, which is common in the Caucasian population [14], that was suggested to protect against environmental toxins that cause neurodegeneration. Data from mouse models show that variant has a neuroprotective effect against toxins that increase the risk of neurodegenerative disease. The review describes current research on the contribution of HFE variants to neurodegenerative disease prognosis in the context of a hormetic model, which is novel for neurodegenerative diseases.

The research by Levi’s group [15] investigates the role of iron in neurodegeneration [16,17], based on the observation that increased brain iron levels often correlate with disease severity. Their work employed induced human pluripotent stem cell-derived astrocytes from an individual with neuroferritinopathy [18]. Iron accumulation is prevalent in this genetic disorder since it is caused by mutations of the ferritin L chain that reduce ferritin’s capacity to accumulate and detoxify iron. Cells from a neuroferritinopathy patient were induced to differentiate into astrocytes, and after 35 days, they showed elevated iron levels that not only triggered ferroptosis [19] but also placed the astrocytes in a reactive state with elevated levels of IL-6, IL-1β, and glutamate, along with changes in morphology, genes, and proteins involved in astrocyte reactivity. Interestingly, by day 60, most of the factors considered showed reversal, except for an increase in senescence and ferroptosis. It was concluded that iron plays a primary role in inducing astrocyte reactivity. Interestingly, astrocyte reactivity was also observed in TfR2 KO mice with brain iron overload [20].

Roetto’s group studied a mouse model in which both Hfe and Transferrin Receptor 2 (TfR2) genes were been inactivated, specifically in macrophages [21]. This is particularly interesting because macrophages play a central role in iron homeostasis by recycling hemoglobin iron [22], and HFE and TfR2 play a key role in the expression of hepcidin, the key regulator of systemic iron homeostasis in mammals [5]. The mice showed a lower splenic iron content, while splenic Ferroportin-1 transcription was significantly increased. Additionally, their bone marrow macrophages showed increased Transferrin Receptor 1 (TfR1) and significantly increased Ferroportin 1 transcript. The mice showed also a significant increase in Erythropoietin (EPO) production. The study shows that Hfe and TfR2 in macrophages regulate hepatic hepcidin production, leading to iron deficiency in aged animals that impairs erythropoiesis, highlighting the extrahepatic role of these two proteins [23,24].

Gozzelino’s group [25] produced an interesting work on the origin of severe malarial anemia (SMA) [26], which often accompanies increased morbidity in malaria caused plasmodium infection [27]. This type of anemia is characterized by ineffective erythropoiesis caused by impaired erythropoietin (EPO) signaling [28]. Their study analyzed a mouse model that was infected and re-infected with plasmodium, showing that re-infection induced higher levels of circulating EPO, while compensatory mechanisms of splenic RBC production were significantly reduced. They also observed immune response activation in the erythropoietic organs of the reinfected animals. An increase in symptom severity in aged mice was correlated with enhanced activation of the immune system, which significantly impaired erythropoiesis. Their work shows a strict correlation between erythropoiesis and immune cells, which ultimately dictates the severity of SMA.

Lipinsky’s group [29] addressed a different subject: severe iron deficiency anemia (IDA) that is common in pigs in the early postnatal period and, if not treated with iron supplementation, may cause stunted growth and increased mortality [30]. It was demonstrated that IDA compromised piglets’ immunocompetence [31]. This iron deficiency is mainly caused by inadequate placental iron transfer from the sow to the fetuses, and it is thought that iron supplementation for pregnant sows might solve the problem. This interesting review provides a critical evaluation of various iron supplementation approaches in pregnant sows for preventing IDA in piglets while avoiding iron toxicity.

In conclusion, this Special Issue provides novel data on the regulation of iron homeostasis in neurodegeneration, malaria, piglets, and some interesting mouse models.
